# Physical Interventions Restore Physical Frailty and the Expression of CXCL-10 and IL-1β Inflammatory Biomarkers in Old Individuals and Mice

**DOI:** 10.3390/biom14020166

**Published:** 2024-01-31

**Authors:** Diego Marcos-Pérez, Sara Cruces-Salguero, Esther García-Domínguez, Marcos J. Araúzo-Bravo, Mari Carmen Gómez-Cabrera, José Viña, Itziar Vergara, Ander Matheu

**Affiliations:** 1 Cellular Oncology Group, Biogipuzkoa Health Research Institute, 20014 San Sebastián, Spain; diego.marcosperez@biodonostia.org (D.M.-P.); sara.crucessalguero@biodonostia.org (S.C.-S.); 2 Freshage Research Group, Faculty of Medicine, Fundación Investigación Hospital Clínico Universitario/Health Research Institute INCLIVA, University of Valencia, 46010 Valencia, Spain; esther.dominguez@uv.es (E.G.-D.); carmen.gomez@uv.es (M.C.G.-C.); jose.vina@uv.es (J.V.); 3 Centro de Investigación Biomédica en Red Fragilidad y Envejecimiento Saludable (CIBERfes), 28029 Madrid, Spain; 4 Computational Biology and Systems Biomedicine, Biodonostia Health Research Institute, 20014 San Sebastián, Spain; mararabra@yahoo.co.uk; 5 IKERBASQUE, Basque Foundation for Science, 48009 Bilbao, Spain; 6 Primary Care Group, Biogipuzkoa Health Research Institute, 20014 San Sebastián, Spain; mariaiciar.vergaramicheltorena@osakidetza.eus

**Keywords:** frailty, inflammation, senescence, physical intervention

## Abstract

Background: Frailty is a geriatric syndrome associated with negative health outcomes that represents a dynamic condition with a potential of reversibility after physical exercise interventions. Typically, inflammatory and senescence markers are increased in frail individuals. However, the impact that physical exercise exerts on inflammatory and senescence biomarkers remains unknown. We assessed the effect of physical intervention in old individuals and mice and determined the expression of inflammatory and senescence markers. Methods: Twelve elderly individuals were enrolled from a primary care setting to a 3-month intervention. Frailty was measured by SPPB and the expression of biomarkers by cytokine array and RT-qPCR. In addition, 12 aged *C57BL/6* mice completed an intervention, and inflammation and senescence markers were studied. Results: The physical intervention improved the SPPB score, reducing frail and pre-frail individuals. This was correlated with a reduction in several pro-inflammatory biomarkers such as IL-6, CXCL-1, CXCL-10, IL-1β, IL-7, GM-CSF as well as *p16^INK4a^* and *p21^CIP1^* senescence markers. Otherwise, the levels of anti-inflammatory biomarker IL-4 were significantly increased. Moreover, the physical intervention in mice also improved their functional capacity and restored the expression of inflammatory (*Il-1β*, *Cxcl-10*, *Il-6*, and *Cxcl-1*) and senescence (*p21^Cip1^*) markers. Additionally, PLSDA and ROC curve analysis revealed CXCL-10 and IL-1β to be the biomarkers of functional improvement in both cohorts. Conclusions: Our results showed that a physical intervention improves physical frailty, and reverses inflammation and senescence biomarkers comprising CXCL-10 and IL-1β.

## 1. Introduction

Frailty is a multidimensional syndrome that gives rise to a vulnerability to stressors and leads to a higher risk of adverse age-related outcomes, such as disability, dependency, comorbidity, hospitalization, and death, in older adults [1]. Frailty is characterized by a reduced functional reserve, impaired adaptive capacity across multiple physiological systems, and increased vulnerability to stressors, which is induced by a complex and multifactorial interaction between genetic, biological, physical, cognitive, psychological, and environmental factors [1,2]. It has become an emerging public health priority [3].

There are several tests, instruments, and scales to assess frailty. The majority of them are based on clinical and functional measures, but each one can evaluate different aspects of frailty and, therefore, each one can identified specific populations and the prevalence of frailty varies based on the test applied [1,4]. The two frailty measurement tools most commonly accepted and used in research are the Frailty Phenotype—which is based on signs of physical decline including poor grip strength, slow walking speed, weight loss, low activity level, and exhaustion—[5] and the Frailty Index, which counts the number of deficits an individual has [6]. However, they have some limitations and are not applied in everyday clinics, especially in a primary care setting. In this context, the use of objective tests that measure physical performance, do not require special equipment, and take a short time to be completed might be preferable [1]. Thus, instruments such as Short Physical Performance Battery (SPPB), a set of physical function tests including standing balance, normal gait speed, and a timed sit-to-stand test, or the FRAIL scale are more commonly used for objectively physical frailty screening [7,8].

Research on biomarkers of frailty is being conducted to complement scales and questionnaires and to better understand the biology of frailty. In this latter point, research on frailty and several scales has been reverse-translated in recent years into mice [9]. Most of the knowledge about the molecular understanding of frailty comes from independent observational studies in humans or mice and indicates that the processes related to aging, such inflammation, senescence, mitochondria and apoptosis, calcium homeostasis, oxidative stress, cytoskeleton and hormones, metabolism, epigenetic changes, or immune response, are altered with frailty [2,10,11]. Among them, inflammatory and senescence biomarkers are likely the ones best linked to frailty status. Inflammageing is a state characterized by a low-grade, chronic, and systemic up-regulation of the inflammatory response in aging, which has been linked to frailty. Thus, increases in the levels of pro-inflammatory chemokines and cytokines, such as interleukin-1 beta (IL-1β), interleukin-6 (IL-6), and tumor necrosis factor-alpha (TNF-α), as well as decreases in the anti-inflammatory cytokines interleukin-4 (IL-4) or interleukin-10 (IL-10), impair the maintenance of immunological homeostasis and immunosenescence and have been detected in frail individuals [2,12,13]. Senescence is a cellular program that typically entails cell cycle arrest and a complex senescence-associated secretory phenotype (SASP) that can be induced by a myriad of intrinsic and extrinsic stresses. The proliferative arrest is controlled by activation of the p16^INK4a^/Rb and p53/p21^CIP1^ tumor suppressor networks, whilst senescent cells express and secrete a mixture of extracellular modulators including pro-inflammatory cytokines, chemokines (CXCLs), and growth factors, which together contribute to aging [14] and age-associated diseases, including frailty [15]. However, the use of anti-inflammatory medications, such as NASIDS, at high frequency is associated with an increased risk of frailty in older individuals [16], and, nowadays, pharmacological treatment is not recommended for the treatment of frailty [17].

Frailty is a previous stage to dependency, and contrary to this, represents a dynamic condition with the potential for reversibility after an intervention. In this regard, different types of interventions such as physical exercise, nutritional diets, cognitive activities, social support, or multicomponent have been confirmed to be effective and can delay or reverse frailty in older individuals [4]. Similarly, several types of interventions also showed efficacy to improve the functional capacity in aged mice [9]. Among them, physical exercise is the best-established intervention to prevent, ameliorate, and reverse frailty status in both humans and mice [18]. In this sense, physical interventions [19] as well as rehabilitation models [20] showed satisfactory results in improving SPPB scores in older adults.

Different meta-analyses and systematic reviews have shown that exercise has a positive impact on reducing inflammation in individuals with chronic and age-related illnesses such as cancer, diabetes, or cardiovascular diseases [21,22]. Similarly, the literature assessing the influence of exercise on inflammatory and senescence biomarkers in frail older adults [2,10,23,24,25,26] and mice [13,27,28] is starting to unravel potential biomarkers and molecular pathways. However, these studies have been completed independently and did not consider samples derived from different tissues. Moreover, the literature assessing these associations in a primary care setting remains to be elucidated. Having this in mind, the aim of this work was to assess the effect of a physical intervention in frail individuals enrolled in a primary care setting, as well as aged mice, and determine the alteration of a set of inflammatory and senescence markers on them.

## 2. Materials and Methods

### 2.1. Primary Care Setting-Enrolled Cohort

Twelve community dwelling older persons (71–82 years) were recruited from a primary care setting in Gipuzkoa (San Sebastián, Spain). Participants completed a physical intervention plan consisting of two supervised training sessions (including strength, flexibility, and aerobic exercises), 1 h per week, for 3 months. More information regarding the cohort, participants, and the intervention can be obtained in [29]. Participants were excluded if they were taking antineoplastic or immunomodulating medication and had any chronic infection or illness. Before and after finishing the physical intervention, participants were evaluated based on Timed Up and Go (TUG) [30] and the Short Physical Performance Battery (SPPB) [31] tests. The adherence rate was 100%, and patients did not suffer any clinical adverse effects. Frailty status was assessed using the SPPB test, and participants were classified as robust (10–12 points), pre-frail (7–9 points), and frail (4–6 points) as previously described in the Vivifrail study [19].

Whole blood samples from all the participants enrolled in this study were collected by venipuncture in ethylenediaminetetraacetic acid (EDTA) tubes (Vacutainer, BD Biosciences, Alphen aan den Rijn, Netherlands) and directly deposited in the Basque Biobank for subsequent processing and storage following their quality and legal procedures (www.biobancovasco.org, accessed on 1 January 2024).

Peripheral blood mononuclear cells (PBMCs) and plasma samples were collected to measure senescence mRNA levels by RT-qPCR and inflammatory markers by Human Cytokine Array C5 (AAH-CYT-5, RayBiotech Inc., Norcross, GA, USA), respectively.

### 2.2. Animal Experimentation

Twenty *C57BL/6J* mice that were twenty months old and male were randomized into two groups: rest (*n* = 8) and trained (*n* = 12). *C57BL/6J* was selected for being largely used in aging studies. The trained group followed a high-intensity interval training protocol consisting of 6 bouts of a running exercise on a treadmill at 100% of their VO_2max_ with 1 min of active rest in between at 50% of their VO_2max_. Mice were exercised 2 days a week for 10 weeks. All animals were evaluated before and after the intervention for different functional parameters (see below). The experimental protocol was approved by the Committee of Ethics in Research of the Faculty of Medicine, University of Valencia.

Body weight: Mice’s body weight was recorded every 7 days using a PB3002 DeltaRange balance scale (Mettler Toledo S.A., Madrid, Spain).

Grip strength: Maximal grip strength was evaluated by using the Grip Strength Meter. The mice were held by the base of the tail and allowed to grasp the “T” drawbar of the apparatus with their front paws for a few seconds. The peak amount of force applied was registered in grams by the apparatus and normalized by the animal’s weight.

Motor coordination: We used the Rotarod to evaluate the animals’ motor coordination. The protocol consisted in increasing the speed of the Rotarod from 4 rpm to 40 rpm in 5 min, and the maximal time that the mice were able to stay on the rod until falling was recorded.

Endurance: A treadmill for mice was used for the measurement of their endurance capacity during an incremental treadmill test. After a warm-up period of 4 min at 6.0 m/min, the treadmill band velocity was increased by 2.4 m/min every 2 min until exhaustion. Total running distance (m) achieved was recorded.

### 2.3. RNA Extraction, cDNA Synthesis, and RT-qPCR

Total RNA from human PBMCs was isolated using QIAamp RNA Blood Mini Kit (QIAGEN Iberia, S.L., Barcelona, Spain) and following the manufacturer’s instructions. In the case of mice skeletal muscle, samples were first homogenized by a tissue-lyser tissue dissociation apparatus with 1 mL TRI Reagent solution and using stainless steel beads. All procedures with RNA were performed in tubes treated for 24 h with diethyl pyrocarbonate for inhibiting RNases. For cDNA synthesis from RNA samples, reverse transcription (RT) was performed by Maxima First Strand cDNA Synthesis Kit (Thermo Fisher Scientific S.L., Bilbao, Spain). The expression levels of the genes of interest were measured with a real-time polymerase chain quantitative reaction (RT-qPCR) using 20 ng cDNA as the reaction template. Reactions were performed in triplicate in CFX384 Touch Real-Time PCR Detection System (Bio-Rad Laboratories, S.A., Madrid, Spain). As an internal control, glyceraldehyde 3-phosphate dehydrogenase (GAPDH) and beta-2-microglobulin (B2M) housekeeping genes were used in the case of human samples, whereas β-actin was used in mice samples. Relative quantification was calculated using the 2^−ΔΔCt^ formula. Results were represented as fold change (FC).

### 2.4. Human Circulating Inflammatory Markers

Plasma samples were used to simultaneously measure 80 different inflammatory markers with the Human Cytokine Array C5. The spot intensity signal was captured and recorded with the iBrightFL1000 imaging system. Densitometry software Image J version 1.53k (National Institute of Health (NIH), Bethesda, MD, USA) was used to perform a numerical comparison of the signal intensities, and RayBiotech Microsoft^®^ Excel-based Analysis Software Tools system was used for each array kit for automatic analysis (www.raybiotech.com, accessed on 1 January 2024).

### 2.5. Statistical Analysis

Results are reported as mean ± standard error of the mean (SEM). Gaussian distribution was assessed with the Shapiro–Wilk test, and paired Student’s *t*-test and the Wilcoxon test were applied to data normally or not normally distributed in the primary care cohort, respectively. The same analysis was performed in the mice cohort but with a non-paired Student’s *t*-test or Mann–Whitney test, depending on normality test results. The intervention effect on clinical variables was assessed using Analysis of Covariance (ANCOVA). Before conducting the test, linearity, homogeneity of regression slopes, and normality assumptions were checked. Partial Least Squares Discriminant Analysis (PLSDA) was used to classify functional changes after the intervention. In the human cohort, groups were defined as individuals who improved after the intervention (*n* = 8) and individuals that did not (*n* = 4), based on SPPB score changes before and after physical exercise. Spot intensity ratio (post-/pre-intervention) was used as the input for the algorithm. In the case of the mice cohort, PLSDA was employed to distinguish between rest and trained groups, and mRNA expression (post-intervention) as well as pre-intervention clinical measurements to adjust the model were used as the input variables. Variable Importance in Projection (VIP) was performed to determine the most important variables in the classification. Variables with higher VIP scores were considered the most relevant ones in the model.

The predictive power of the individual biomarkers was assessed through ROC curves. PLSDA and ROC curves were performed through *mixOmics* and pROC packages, respectively, in RStudio software (version 4.2.2, URL: https://www.r-project.org/, accessed on 1 January 2024). Statistical analysis was performed using GraphPad Prism version 9.4.1 (Boston, MA, USA). Statistical significance was established at *p* < 0.001 (***), indicating very strong evidence; *p* < 0.01 (**), strong evidence; *p* < 0.05 (*), moderate evidence; and *p* < 0.1 (≠), weak evidence or a trend.

## 3. Results

### 3.1. Intervention with Physical Exercise on Community Dwelling Older Persons

**Figure 1:**
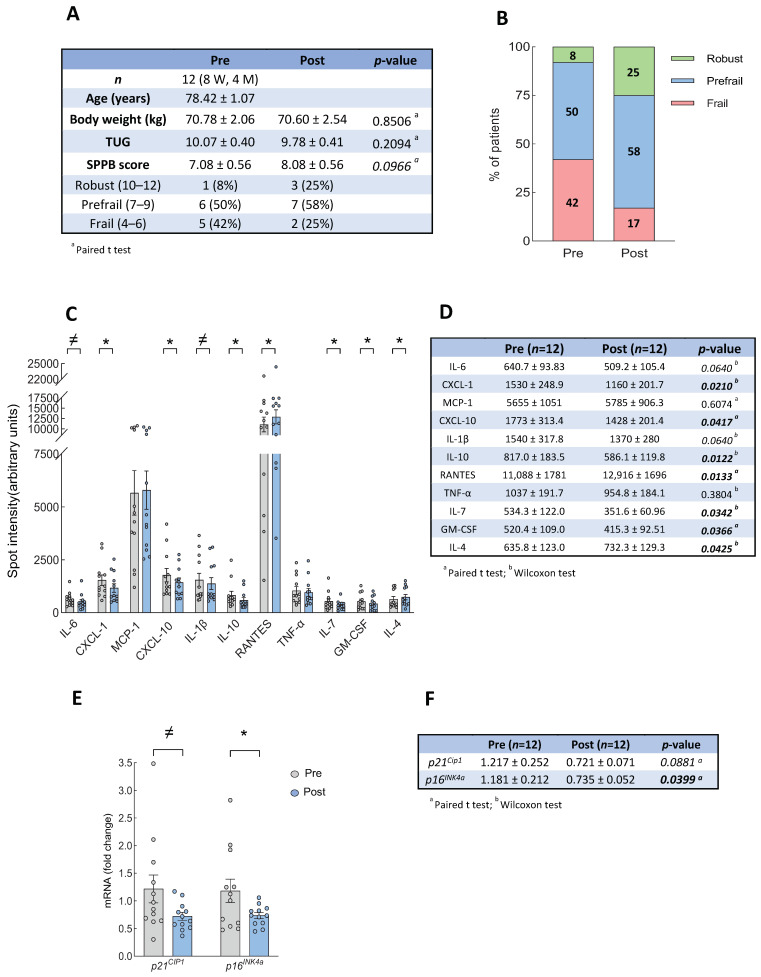
Analysis of clinical data and inflammatory and senescence markers in community dwelling individuals enrolled in primary care setting. (**A**) Description of participants (*n* = 12) before and after 12-week exercise training intervention. (**B**) Changes of percentage of individuals in each frailty category before and after physical intervention based on SPPB score. (**C**) Levels of plasma circulating inflammatory genes differentially expressed in basal condition (pre) and after exercise training (post). (**D**) Data as mean ± standard error of mean (SEM), and *p*-values of inflammatory mediators. (**E**) PBMCs mRNA expression levels of senescence markers. (**F**) Data as mean ± SEM, and *p*-values of senescence markers. ^a^ paired *t*-test, ^b^ Wilcoxon test. Bold numbers represent statistical significance (*p* < 0.05), and italic numbers represent tendency (*p* < 0.1). * *p* < 0.05; ≠ *p* < 0.1.

Individuals that completed the intervention showed a weak tendency of improvement in the SPPB score (*p* = 0.09), with an improvement in SPPB in 8 out of 12 cases, with a mean increase of 1 point in the SPPB score, from 7.08 ± 0.56 in basal conditions to 8.08 ± 0.56 points after the intervention (Figure 1A). In line with this, the percentage of individuals classified as frail decreased (from 42% to 17%), whereas the pre-frail and robust increased, from 50 to 58% in pre-frails and from 8 to 25% in robust individuals (Figure 1B). These changes were also explored specifically for each gender, with men showing a notable improvement in SPPB after the intervention, although there were only four cases (Supplementary File S1). Similarly, the Time Up and Go (TUG) score did not reach statistical significance but the result was improved in 7 out of 12 cases (Figure 1A). On the contrary, participants did not show significant weight alterations (loss or gain) during the intervention period (Figure 1A). With the group and intervention being characterized, we measured the expression of a battery of 80 inflammatory and SASP biomarkers in blood samples pre- and post-intervention and found an alteration in the expression of a few of them after the intervention (Supplementary File S2). In particular, we found a significant reduction of several pro-inflammatory biomarkers such as IL-6, IL-1β, CXCL-1, CXCL-10, IL-7, and GM-CSF (Figure 1C,D). Aside from this, the levels of the anti-inflammatory cytokine IL-4 were significantly increased, whereas IL10 and RANTES (CCL5) decreased (Figure 1C,D). We also studied the mRNA expression of the senescence markers *p16^INK4a^* and *p21^CIP1^* with q-RTPCR after the physical intervention and we detected a reduced expression in both cases (Figure 1E,F). These results suggest that the expression of several inflammation and senescence biomarkers are restored after physical exercise interventions. In order to further extend these results, we completed association analysis with the different biomarkers, which showed that the reversion of physical performance on the SPPB test was positively correlated with changes in the majority of the biomarkers. Notably, there was a positive correlation with IL-1β and CXCL-10 levels in 92% and 83% of the individuals, respectively, and we found an improvement in IL-6, GM-CSF, TNF-α, *p16^INK4a^*, and *p21^CIP1^* levels in 67% of the participants (Supplementary File S3).

**Figure 2:**
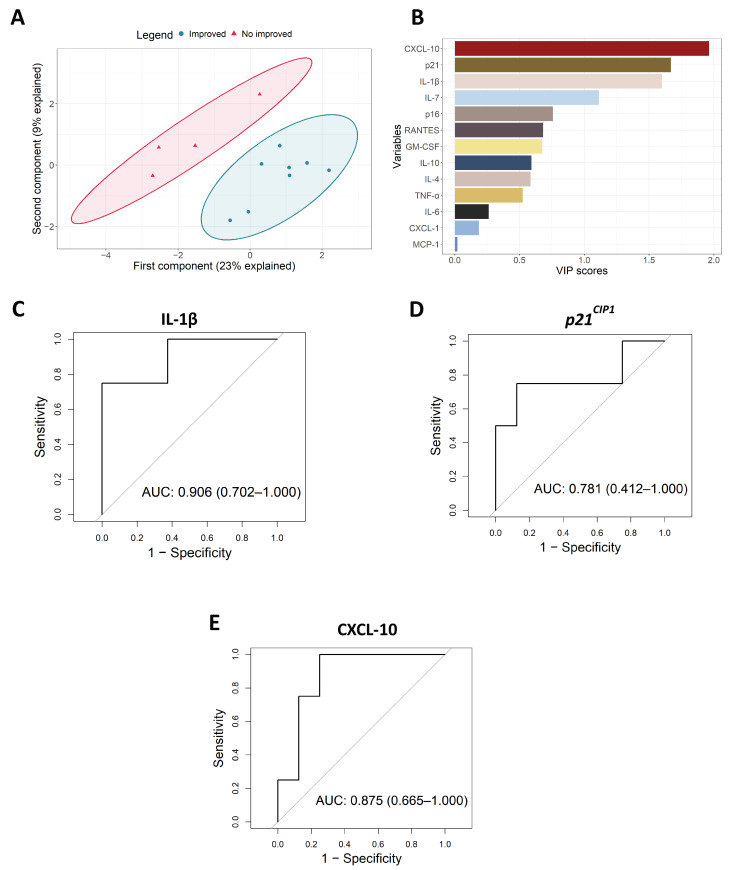
PLSDA analysis and ROC curves of primary care setting cohort. (**A**) PLSDA model classifying participants based on inflammatory and senescence measurements. (**B**) VIP score rank of markers’ importance implicated in PLSDA classification. (**C**–**E**) IL-β, *p21^CIP1^*, and CXCL-10 ROC curves, respectively.

PLSDA analysis was performed to classify participants’ functional changes by using only inflammatory and senescence changes as the inputs in the model. We defined two groups based on the changes in the SPPB score after the intervention: individuals who improved and individuals who did not. PLSDA resultant latent variables were able to perfectly distinguish between individuals who improved or did not improve their functional status after the intervention, with the first and second components explaining 23% and 9% of the variance, respectively (Figure 2A). In order to assess the importance of each biomarker in the process of classification, the VIP score was computed. The three biomarkers that most contributed to the model were CXCL-10, *p21^CIP1^*, and IL-1β, with a VIP score higher than 1.5 (Figure 2B). This is in agreement with the results obtained in the association analysis (see Supplementary File S3). To further characterize the impact of the different biomarkers, ROC curves were performed for inflammatory and senescence biomarkers. The most accurate ROC curves were those of IL-1β, *p21^CIP1^*, and CXCL-10, respectively with areas under the curve (AUC) of 0.906 (0.702–1.000), 0.781 (0.412–1.000), and 0.875 (0.665–1.000), respectively (Figure 2C–E and Supplementary File S4). This analysis reinforces the VIP score results and postulates these three genes as the most robust biomarkers associated with physical exercise interventions.

### 3.2. Intervention with Physical Exercise on Aged Mice

**Figure 3:**
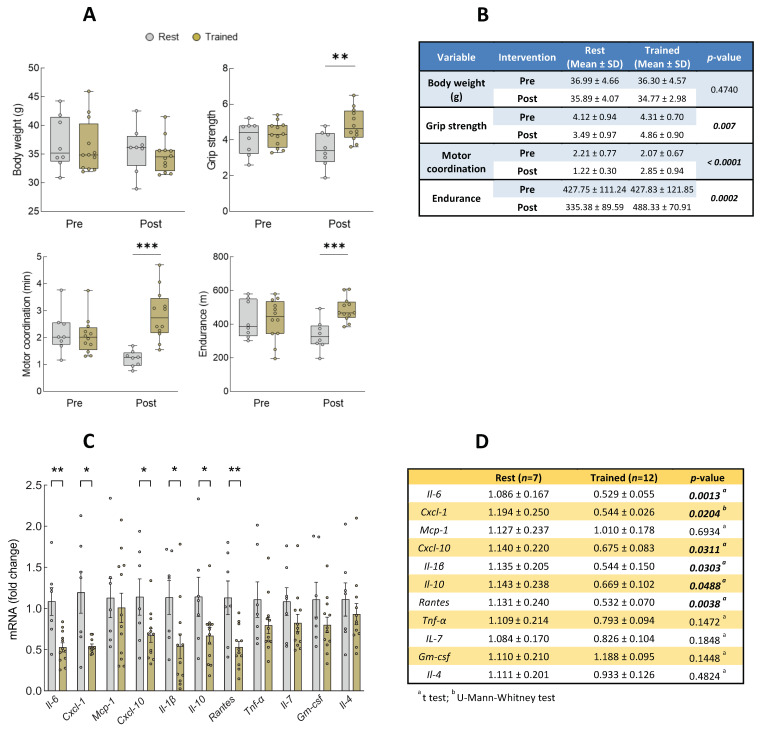

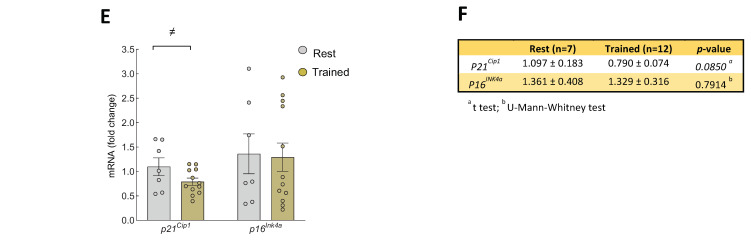
Analysis of functional parameters and mRNA levels of inflammatory and senescence markers in mice cohort. (**A**) Description of 23-month male *C57BL/6* mice cohort, rested group (*n* = 8), and trained group (*n* = 12) before and after 10-week physical intervention. (**B**) ANCOVA analysis of clinical data. (**C**) mRNA expression levels of inflammatory mediators. (**D**) Data as mean ± standard error of mean (SEM), and *p*-values of inflammatory mediators. (**E**) mRNA expression levels of senescence markers. (**F**) Data as mean ± SEM, and *p*-values of senescence markers. ^a^ *t*-test, ^b^ U-Mann–Whitney test. Bold numbers represent statistical significance (*p* < 0.05), and italic numbers represent tendency (*p* < 0.1). *** *p* < 0.001; ** *p* < 0.01; * *p* < 0.05; ≠ *p* < 0.1.

**Figure 4:**
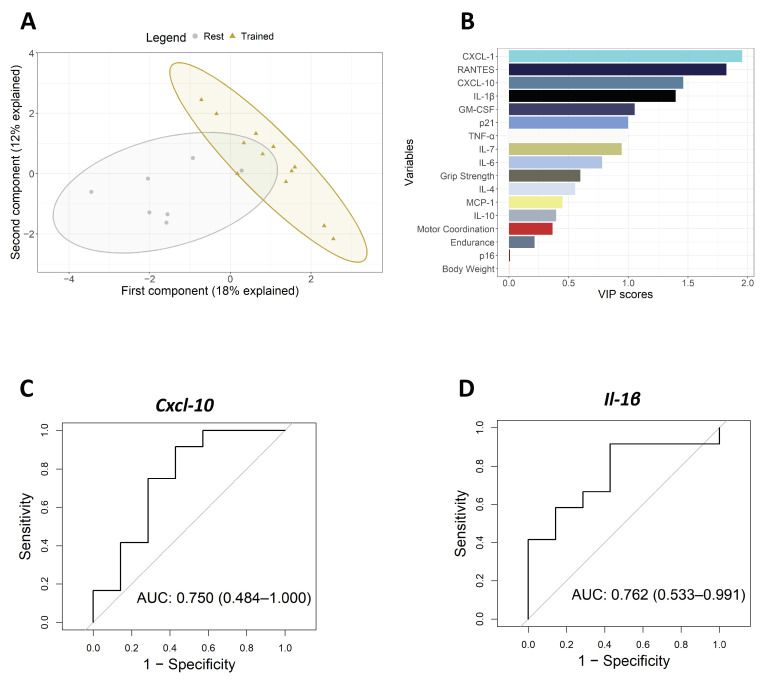
PLSDA analysis and ROC curves of mice cohort. (**A**) PLSDA model classifying mice based on inflammatory, senescence, and clinical measurements. (**B**) VIP score rank of variable importance implicated in PLSDA classification. (**C**,**D**) *Cxcl-10* and *Il-1β* ROC curves, respectively.

To further validate the effect of inflammation and senescence after the physical intervention, we moved our focus to the aged mice. There, we studied the expression of the altered set of inflammatory biomarkers in human samples as well as *p16^INK4a^* and *p21^CIP1^* senescence markers in muscle samples after 10 weeks of exercise and compared them to aged non-exercised mice. First, we confirmed that the mice before the intervention did not have differences between the rest and trained groups in their functional characteristics (Supplementary File S5A). When comparing the effects of the intervention in both groups, we observed that non-exercised mice suffered a significant functional decline after these 10 weeks in both motor coordination and endurance tests (Supplementary File S5B). However, we detected a significant functional improvement in trained mice compared with the rested mice for grip strength, motor coordination, and endurance functional parameters after the intervention. The Analysis of Covariance (ANCOVA) verified that the physical improvement was exclusively due to the exercise effect (Figure 3A,B). At a molecular level, we detected a significant decrease in the mRNA level of the pro-inflammatory mediators *Il-1β*, *Cxcl-10*, *Il-6*, and *Cxcl-1*, as well as in the senescence marker *p21^Cip1^*, on trained mice after the physical intervention compared with the control group (Figure 3C–F). In this case, we performed PLSDA to see if the expression of the biomarkers after the intervention, along with the clinical measurements pre-intervention, was able to distinguish between the intervention and control groups. Indeed, the resultant latent variables were almost capable of perfectly differentiating between the rest and trained mice groups, with the first and second components explaining 18% and 12% of the variance, respectively (Figure 4A). Coherently with our human cohort results, *Cxcl-10* and *Il-1β* were two of the four most important mediators in the model, with VIP score values close to 1.5 (Figure 4B). ROC curves were performed for all biomarkers, obtaining AUCs of 0.762 (0.533–0.991) and 0.750 (0.484–1.000) for *Il-1β*, and *Cxcl-10*, respectively (Figure 4C,D, Supplementary File S6). These results highlight *Il-1β* and *Cxcl-10* as the biomarkers associated with physical exercise interventions in humans but also in animal models.

## 4. Discussion

Exercise is the most beneficial and minimally invasive intervention to counteract the progressive physiological and functional decline associated with frailty in older adults [18]. The molecular mechanisms underlying muscle activity and whether interventions reverse or alter inflammation and senescence biomarkers is currently an area of intense research, but displays contradictory results in some cases [32]. These discrepancies in results may be due to the heterogeneity in aspects such as exercise type (e.g., resistance vs. aerobic), protocols (intervention duration, intensity, etc.), and the demographic variables employed (e.g., age, sex, healthy state of participants, and the presence of diseases, among others). In this regard, our results showed functional improvements in both community-dwelling older individuals and aged mice after two different exercise interventions. In the human pilot study, the intervention group displayed a mean increase of 1 point on the SPPB scale over the control group, which is within the range of being significantly different, and is considered to be 1 point in relation to the SPPB scale [33]. On the other hand, the trained mice presented significant improvements in grip strength, motor coordination, and endurance functional parameters after the intervention, which are three important factors used to measure frailty [9]. Both studies confirmed the benefits of the interventions on the health status, mainly comprising physical activity, of their respective subjects.

Functional improvements were associated with a general reversion of an inflammatory panel, including pro- and anti-inflammatory cytokines, chemokines, and senescence biomarkers in both cohorts. Overall, a significant anti-inflammatory effect of exercise was found in IL-6, CXCL-1, IL-1β, CXCL-10, and p21^CIP1^ levels in both cohorts. In line with our work, independent groups using different types of exercise, including resistance, aerobic, and combined protocols, showed to have an effect on different inflammation markers by lowering circulating IL-6 levels—the cytokine reaction that seemed more common and relevant in healthy older adults [32]. Moreover, and related to senescence, a recent work from Englund and colleagues reported that a 12-week exercise program reduces the expression of p16^INK4A^ and p21^CIP1^, which are components of the cGAS-STING pathway (e.g., cGAS, IFN-γ, and TNF-α), as well as the mediators of circulating SASP biomarkers in older adults [25]. In mice, few articles suggest that pro-inflammatory cytokines and the burden of senescence may be implicated in frailty, muscle atrophy, and/or functional declines [28,34]. Additionally, the impact of different interventions (exercise, dietary supplements/modifications, and pharmacological treatments) to treat frailty regarding circulating cytokine and chemokine levels in mouse models is starting to be studied in detail [13]. Thus, independent studies reported significant mRNA increases in *Il-1α*, *Il-1β*, *Il-6*, and *Tnf-α* inflammatory biomarkers as well as *p16^INK4A^* and *p21^CIP1^* in aged muscle compared to young ones [35,36]. We also observed an increased RANTES expression and a reduced expression of IL-10, which are generally part of the pro- and anti-inflammatory response, respectively. However, the literature related to their expression in frailty and after physical interventions in older adults showed controversial results [12,13]. In relation to RANTES, most of the studies developed that commonly tested humans and mice showed no significant differences in its circulating levels in frail individuals [13]. In the case of IL-10, its anti-inflammatory role on age-related diseases and frailty has been extensively reported, and several studies found increased levels of this cytokine with different types of exercise interventions of different extensions. However, others did not detect any increases of this cytokine in older adults [24,27,37]. It is likely that frailty reflects the disruption in the balance between anti- and pro-inflammatory cytokines instead of the changes in specific cytokines [38]. Additionally, the interventions resemble the combat between the activation of both pro- and anti-inflammatory mechanisms to attenuate frailty, with activations or reductions depending on the intensity and duration of the intervention, as well as participants’ characteristics such as age or gender. It is also likely that the relevance of the different pro- and anti-inflammatory cytokines and chemokines for frailty restoration is not equal. In this respect, a recent study revealed that the trajectories of IL-10 and RANTES concentrations over the previous 20 years are not within the most important ones for predicting frailty [39]. Overall, our data, together with these results, reinforce inflammation and cellular senescence as being the contributing mechanisms of frailty and age-associated functional decline and the potential for physical activity to attenuate and/or partially reverse their critical drivers.

The heterogeneity of the inflammatory network, the senescence process, and the distinct cellular and molecular events involved in them reflect the complexity to associate individual mediators and physiological functions in epidemiological and in vivo studies. Although IL-6 is probably the currently leading biomarker across humans and mice to study inflammageing and frailty, in our study, the restoration was the strongest in the case of CXCL-10, IL-1β, and p21^CIP1^, with them showing significant associations with functional recovery in older individuals, whilst *Cxcl-10* and *Il-1β* showed a robust association with training differences in the mice cohort. In addition, these two markers showed the strongest potential as biomarkers of functional improvement, based on the PLSDA models and ROC curve results. Thus, these results show promise for CXCL-10 and IL-1β as potential translational biomarkers across humans and mice to study frailty and functional improvement after interventions that utilize physical exercise as their modality.

Chemokine C-XC motif ligand 10 (CXCL-10), also known as interferon gamma-induced protein (IP-10), is a multifunctional secreted factor that regulates several biological processes, such as chemotaxis, cell proliferation, and apoptosis. This pro-inflammatory molecule is a protein produced and secreted by a series of cells, including those in skeletal muscle [40], and different studies reported its increases in human serum with age [41,42,43]. Moreover, higher levels of CXCL-10 have also been described in frail individuals; this frailty-associated up-regulation was detected to be highly correlated with IL-6 elevation [44]. In regard to interventions or anti-aging strategies, it has been shown that age-related increases in CXCL-10 levels were counteracted by 8 weeks of an exercise intervention in 413 healthy adults [45], as well as by several interventions targeting age in mice, including exercise training [46,47], caloric restriction [48], or drug interventions using resveratrol or metformin [49,50]. Importantly, CXCL-10 is within the few inflammatory biomarkers that showed a similar relationship in humans and mice in relation to frailty, having been postulated as a promising biomarker of frailty, although still with limited evidence [13]. On the other hand, interleukin-1β (IL-1β) is a potent pro-inflammatory cytokine that is a key mediator of the host defense inflammatory response. IL-1β levels increase with frailty status in humans [51], and also in the frail mouse model IL-10^tm/tm^ [52], but the effect of exercise on IL-1β levels in healthy older adults is not clear yet. A recent review and meta-analysis [53] found 12 studies with 18 intervention groups that investigated the effects of regular exercise on the concentrations of IL-1β in the peripheral blood of older adults. Although they reported a significant decline in IL-1β levels after different intervention programs, only one study [54] was performed in healthy older adults with no significant IL-1β alteration after a 12-week exercise program. In summary, these studies reinforce the potential of CXCL-10 and IL-1β as the biomarkers for frailty in exercise-based interventions in pre-clinical and clinical research, but additional works characterizing these relationships would be helpful in terms of further confirmation. Thus, our study finds similar results in human circulating inflammatory mediators, and on tissue-specific mice skeletal muscle after a physical intervention, which is in line with the evidence that exercise combats the detrimental effects of inflammation on skeletal muscle and other physiological systems [55].

The main limitation of our study is the limited number of participants. Another one is that we did not have a control group in our human study. We tried to solve these limitations by making parallel comparisons between patients’ physical improvement and inflammatory–SASP–senescence outcomes. In this sense, PLSDA analysis showed that biomarkers could be related to a physical improvement in patients and mice cohorts. The latter cohort has functional data from before and after the exercise program, demonstrating that the inflammatory–senescence alterations found in this cohort came from this functional improvement. Additionally, the interventions were different, and the samples were obtained from different tissues. In this sense, the studies of biomarkers after exercise interventions in humans were completed in blood samples that we tried to complement with skeletal muscle samples, since frailty is a multidimensional syndrome and muscles are a critical target regarding the relation of physical frailty with sarcopenia [17].

## 5. Conclusions

Overall, our results revealed an alteration in the pattern of different inflammatory mediators and senescence markers in older human and mice cohorts after different physical interventions on systemic (blood samples) and local (skeletal muscle samples) tissue samples. Among them, CXCL-10 and IL-1β were the two mediators that could be outlined as the strongest potential biomarkers of functional improvement in physically frail subjects.

## Data Availability

No new data were created or analyzed in this study. Data sharing is not applicable to this article.

## Supplementary Materials

The following supporting information can be downloaded at: https://www.mdpi.com/article/10.3390/biom14020166/s1, Supplementary File S1. Description of Primary Care setting cohort according to gender before and after 12-week exercise training intervention; Supplementary File S2. Data as mean ± standard error of mean (SEM), and p-values of the 80 inflammatory mediators analyzed in plasma human samples; Supplementary File S3. Association analysis comparing functional (improve/worsen) and biomarkers (increase/decrease) changes in Primary Care cohort; Supplementary File S4. ROC curves of inflammatory and senescence markers analyzed in Primary Care setting cohort; Supplementary File S5. Analysis of clinical data in mice cohort. (A) Comparison of functional parameters before the intervention in trained vs. rest group. (B) Comparison of functional parameters in rest mice before and after the intervention; Supplementary File S6. ROC curves of inflammatory and senescence markers analyzed in mice cohort.
