# Supplementary material for: Physical Interventions Restore Physical Frailty and the Expression of CXCL-10 and IL-1β Inflammatory Biomarkers in Old Individuals and Mice
Source: Biomolecules. 2024 Jan 31;14(2):166. doi: 10.3390/biom14020166 (PMC10886745; doi:10.3390/biom14020166)
Supplement: Supplementary file 1 [file biomolecules-14-00166-s001.zip › biomolecules-2711353-supplementary.pdf]

	Pre		Post	
	Women (*n* = 8)	Men (*n* = 4)	Women (*n* = 8)	Men (*n* = 4)
**Age (years)**	78.63 ± 1.39	78 ± 1.87		
**Body weight (kg)**	69.19 ± 2.22	74.23 ± 4.24	69.01 ± 3.07	73.78 ± 4.67
**TUG**	10.53 ± 0.48	9.15 ± 0.57	10.07 ± 0.48	9.2 ± 0.77
**SPPB score**	6.75 ± 0.67	7.75 ± 1.03	7.5 ± 0.65	9.25 ± 0.85
Robust (10-12)	0 (0%)	1 (25%)	1 (12.5%)	2 (50%)
Prefrail (7-9)	5 (62.5%)	1 (25%)	5 (62.5%)	2 (50%)
Frail (4-6)	3 (37.5%)	2 (25%)	2 (25%)	0 (0%)

Supplementary File S1. Description of Primary Care setting cohort according to gender before and after 12-week exercise training intervention.

Marker	Pre	Post	*p*-value
**ENA-78**	1717 ± 229.9	1815 ± 263.1	0.5186[a]
**G-CSF**	590.1 ± 68.86	479.7 ± 64.85	0.0989[b]
**GM-CSF**	520.4 ± 109	415.3 ± 92.51	0.0366[b]
**GRO**	2291 ± 446.5	2801 ± 549.3	0.1302[b]
**GRO-alpha**	1530 ± 248.9	1160 ± 201.7	0.021[a]
**I-309**	786.5 ± 209.2	1002 ± 222	0.0015[a]
**IL-1alpha**	939.3 ± 249.8	910.3 ± 194.8	0.9697[a]
**IL-1beta**	1540 ± 317.8	1370 ± 280	0.064[a]
**IL-2**	581.5 ± 115.4	603.2 ± 121.9	0.8501[a]
**IL-3**	1856 ± 380.1	1846 ± 357.8	>0.9999[a]
**IL-4**	635.8 ± 123	732.3 ± 129.3	0.0425[a]
**IL-5**	1319 ± 274.4	1381 ± 260.4	0.5693[a]
**IL-6**	640.7 ± 93.83	509.2 ± 105.4	0.064[a]
**IL-7**	534.3 ± 122	351.6 ± 60.96	0.0342[a]
**IL-8**	2124 ± 487.1	2199 ± 521	0.3804[a]
**IL-10**	817 ± 183.5	586.1 ± 119.8	0.0122[a]
**IL12-p40**	1304 ± 224.6	1330 ± 233.5	>0.999[a]
**IL-13**	406.8 ± 51.17	468.4 ± 55.15	0.2334[a]
**IL-15**	939.8 ± 191.8	992.4 ± 170.4	0.4697[a]
**IFN-gamma**	1362 ± 117	1361 ± 132.8	0.985[b]
**MCP-1**	6061 ± 1062	6199 ± 1008	0.6377[a]
**MCP-2**	1465 ± 371.8	1379 ± 322	0.3804[a]
**MCP-3**	793.3 ± 157.1	721.6 ± 151.8	0.6772[a]
**M-CSF**	1922 ± 417.2	1840 ± 406.5	0.9097[a]
**MDC**	1874 ± 278	1751 ± 242.6	0.6564[b]
**MIG**	5056 ± 666.4	4589 ± 542.2	0.463[b]
**MIP-1 beta**	2296 ± 531.8	2163 ± 448.7	0.4238[a]
**MIP-1-delta**	5157 ± 624.3	5428 ± 695.8	0.3385[b]
**RANTES**	11,088 ± 1781	12,916 ± 1696	0.0133[b]
**SCF**	1239 ± 281.2	1159 ± 237.6	0.2661[a]
**SDF-1**	2469 ± 844.5	2266 ± 758.2	0.4238[a]
**TARC**	1217 ± 191.5	1095 ± 128.8	0.2112[b]
**TGF-beta 1**	689.3 ± 221.9	581.3 ± 159.8	0.2334[a]
**TNF-alpha**	1037 ± 191.7	954.8 ± 184.1	0.3804[a]
**TNF-beta**	1058 ± 253.9	1012 ± 243	0.8501[a]
**EGF**	2619 ± 336.4	2931 ± 397.9	0.0782[a]
**IGF-1**	3308 ± 334.8	2830 ± 306.1	0.2231[b]
**Angiogenin**	17,941 ± 984.2	18,099 ± 1558	0.8605[b]
**Oncostatin M**	1579 ± 303.5	1826 ± 297.6	0.1514[a]
**TPO**	889.3 ± 213.9	1041 ± 258.4	0.0425[a]
**VEGF**	1068 ± 277.9	1022 ± 234	0.791[a]
**PDGF-BB**	2624 ± 567.6	2517 ± 416.6	0.8501[a]
**Leptin**	8658 ± 1934	9398 ± 1825	0.3394[a]
**BDNF**	5719 ± 1000	5736 ± 815.4	0.9657[b]
**BLC**	1068 ± 257.8	920.9 ± 173.7	0.2661[b]
**CK beta 8-1**	867.7 ± 210.1	732.2 ± 141.7	0.2036[a]

Marker	Pre	Post	*p*-value
**Eotaxin**	2490 ± 495.3	2351 ± 304.6	0.585[b]
**Eotaxin-2**	1615 ± 351.7	1493 ± 218.8	0.5026[b]
**Eotaxin-3**	1122 ± 148.5	1033 ± 102.8	0.5186[a]
**FGF-4**	1065 ± 271.5	1027 ± 216.7	0.9697[a]
**FGF-6**	405.9 ± 35.43	419.8 ± 43.88	0.5518[b]
**FGF-7**	663.8 ± 187	593.6 ± 158	0.3013[a]
**FGF-9**	1471 ± 363.7	1365 ± 276.8	0.6221[a]
**Flt-3 Ligand**	1508 ± 413.3	1288 ± 297.9	0.5825[a]
**Fractalkine**	1231 ± 307.9	936.7 ± 193.7	0.0737[a]
**GCP-2**	1084 ± 300	839.2 ± 175.9	0.1294[a]
**GDNF**	1316 ± 248.2	1151 ± 131.8	0.4697[a]
**HGF**	850.9 ± 184.9	754.4 ± 119.8	0.6772[a]
**IGFBP-1**	3923 ± 931.2	3596 ± 625	0.4532[b]
**IGFBP-2**	8487 ± 1119	8859 ± 722.3	0.5876[b]
**IGFBP-3**	1495 ± 436.8	1715 ± 383.7	0.2334[a]
**IGFBP-4**	3087 ± 1180	3126 ± 1185	0.9097[a]
**IL-16**	990.5 ± 233.6	929.9 ± 190	0.9097[a]
**IP-10**	1773 ± 313.4	1428 ± 201.4	0.021[a]
**LIF**	1165 ± 296.3	954.9 ± 201.5	0.1763[a]
**LIGHT**	1126 ± 251.6	898.6 ± 157	0.1099[a]
**MCP-4**	793.6 ± 207.5	645 ± 121.9	0.5044[a]
**MIF**	1282 ± 357.6	1124 ± 313	0.6221[a]
**MIP-3-alpha**	675.3 ± 126.5	609.2 ± 103.8	0.3577[b]
**NAP-2**	5909 ± 837.7	5354 ± 723.8	0.1011[b]
**NT-3**	1418 ± 200.8	1383 ± 147.3	0.9097[a]
**NT-4**	932.8 ± 238.5	952.5 ± 218.2	0.7183[a]
**Osteopontin**	11,903 ± 1633	12,746 ± 1223	0.255[b]
**Osteoprotegerin**	1083 ± 286.4	1014 ± 201.2	0.7334[a]
**PARC**	3490 ± 961.7	3390 ± 863	0.6221[a]
**PIGF**	681.2 ± 160	573.5 ± 115.9	0.1699[a]
**TGF- b 2**	1124 ± 421.6	867.2 ± 293.2	0.3804[a]
**TGF- b 3**	1084 ± 252.6	851.8 ± 125.3	0.2661[a]
**TIMP-1**	7015 ± 963.2	6121 ± 766.6	0.2451[b]
**TIMP-2**	12,102 ± 856.8	12,010 ± 702.8	>0.999[a]

Supplementary File S2. Data as mean ± standard error of mean (SEM), and p-values of the 80 inflammatory mediators analyzed in plasma human samples. a paired t test, b Wilcoxon test.

IL-6			
SPPB\Marker	Up	Down	Total %
Improved	2/12	**6/12**	**67%**
Worsen	**2/12**	2/12	

TNF-α			
SPPB\Marker	Up	Down	Total %
Improved	2/12	**6/12**	**67%**
Worsen	**2/12**	2/12	

CXCL-1			
SPPB\Marker	Up	Down	Total %
Improved	1/12	**7/12**	**58%**
Worsen	**-**	4/12	

IL-7			
SPPB\Marker	Up	Down	Total %
Improved	2/12	**6/12**	**58%**
Worsen	**1/12**	3/12	

MCP-1			
SPPB\Marker	Up	Down	Total %
Improved	4/12	**4/12**	**50%**
Worsen	**2/12**	2/12	

GM-CSF			
SPPB\Marker	Up	Down	Total %
Improved	1/12	**7/12**	**67%**
Worsen	**1/12**	3/12	

CXCL-10			
SPPB\Marker	Up	Down	Total %
Improved	1/12	**7/12**	**83%**
Worsen	**3/12**	1/12	

IL-4			
SPPB\Marker	Up	Down	Total %
Improved	**6/12**	2/12	**58%**
Worsen	3/12	**1/12**	

IL-1β			
SPPB\Marker	Up	Down	Total %
Improved	-	**8/12**	**92%**
Worsen	**3/12**	1/12	

p21			
SPPB\Marker	Up	Down	Total %
Improved	1/12	**7/12**	**67%**
Worsen	**1/12**	3/12	

IL-10			
SPPB\Marker	Up	Down	Total %
Improved	**5/12**	3/12	**50%**
Worsen	3/12	**1/12**	

p16			
SPPB\Marker	Up	Down	Total %
Improved	2/12	**6/12**	**67%**
Worsen	**2/12**	2/12	

RANTES			
SPPB\Marker	Up	Down	Total %
Improved	**7/12**	1/12	**58%**
Worsen	4/12	**-**	

Supplementary File S3. Association analysis comparing functional (improve/worsen) and biomarkers (increase/decrease) changes in Primary Care cohort.

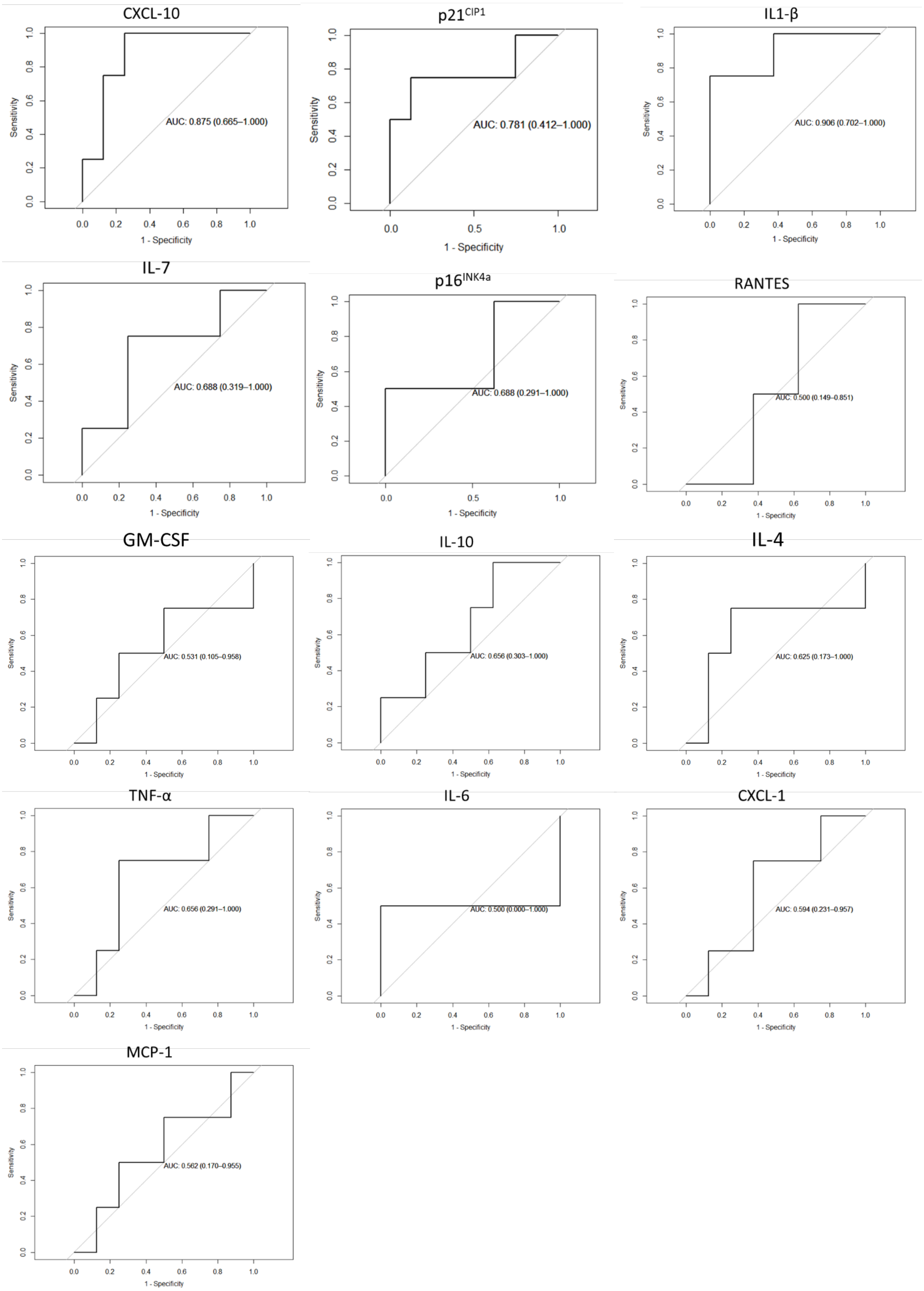

Supplementary File S4. ROC curves of inflammatory and senescence markers analyzed in Primary Care setting cohort.

A

Variable	Rest (Mean ± SD)	Trained (Mean ± SD)	*p*-value
**Body weight**	36.99 ± 4.66	36.30 ± 4.57	0.8506
**Grip strength**	4.12 ± 0.94	4.31 ± 0.70	0.6217
**Motor coordination**	2.21 ± 0.77	2.07 ± 0.67	0.6917
**Endurance**	427.75 ± 111.24	427.83 ± 121.85	0.9988

B

Variable	Pre (Mean ± SD)	Post (Mean ± SD)	*p*-value
**Body weight**	36.99 ± 4.66	35.89 ± 4.07	0.3663
**Grip strength**	4.12 ± 0.94	3.49 ± 0.97	*0.0967*
**Motor coordination**	2.21 ± 0.77	1.22 ± 0.30	***0.0027***
**Endurance**	427.75 ± 111.24	335.38 ± 89.59	***0.0038***

Supplementary File S5. Analysis of clinical data in mice cohort. (**A**) Comparison of functional parameters before the intervention in trained vs rest group. (**B**) Comparison of functional parameters in rest mice before and after the intervention. Bold numbers represent statistical significance ($p < 0.05$), and italic numbers represents a tendency ($p < 0.1$).

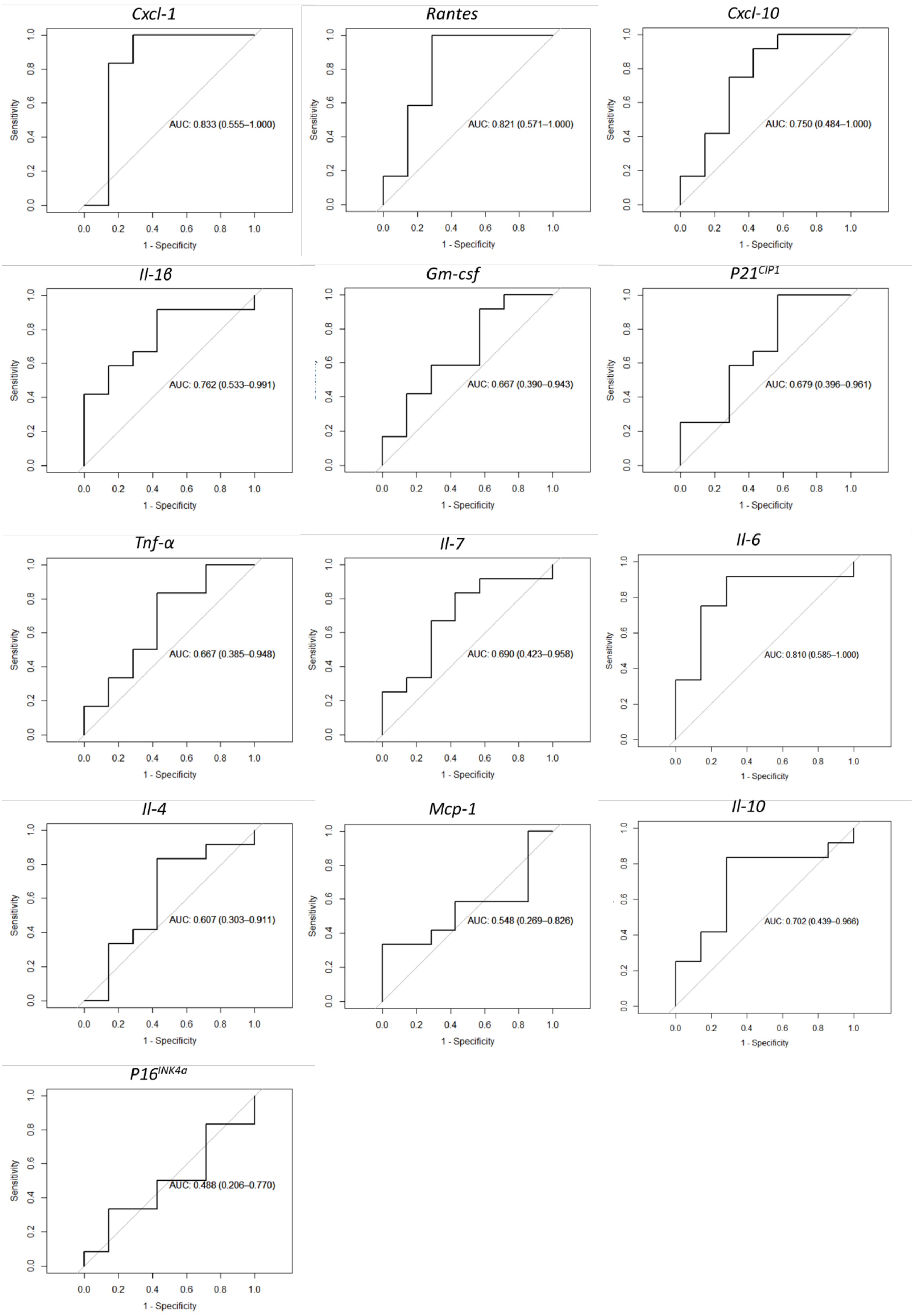

Supplementary File S6. ROC curves of inflammatory and senescence markers analyzed in mice cohort.
